# The destruction of mucosal barriers, epithelial remodeling, and impaired mucociliary clearance: possible pathogenic mechanisms of *Pseudomonas aeruginosa* and *Staphylococcus aureus* in chronic rhinosinusitis

**DOI:** 10.1186/s12964-023-01347-2

**Published:** 2023-10-30

**Authors:** Zahra Chegini, Milad Noei, Jaber Hemmati, Mohammad Reza Arabestani, Aref Shariati

**Affiliations:** 1 Department of Microbiology, School of Medicine, Hamadan University of Medical Sciences, Hamadan, Iran; 2 Department of Genetics, Faculty of Advanced Science and Technology, Tehran Medical Sciences, Islamic Azad University, Tehran, Iran; 3 Student Research Committee, Khomein University of Medical Sciences, Khomein, Iran

**Keywords:** CRS, *Staphylococcus aureous*, *Pseudomonas aeruginosa*, Mucociliary clearance, Epithelial remodeling, Mucosal barriers

## Abstract

Chronic rhinosinusitis (CRS) is a pathological condition characterized by persistent inflammation in the upper respiratory tract and paranasal sinuses. The epithelium serves as the first line of defense against potential threats and protects the nasal mucosa. The fundamental mechanical barrier is formed by the cell-cell contact and mucociliary clearance (MCC) systems. The physical-mechanical barrier is comprised of many cellular structures, including adhesion junctions and tight junctions (TJs). To this end, different factors, such as the dysfunction of MCC, destruction of epithelial barriers, and tissue remodeling, are related to the onset and development of CRS. Recently published studies reported the critical role of different microorganisms, such as *Staphylococcus aureus* and *Pseudomonas aeruginosa*, in the induction of the mentioned factors. Bacteria could result in diminished ciliary stimulation capacity, and enhance the chance of CRS by reducing basal ciliary beat frequency. Additionally, bacterial exoproteins have been demonstrated to disrupt the epithelial barrier and induce downregulation of transmembrane proteins such as occludin, claudin, and tricellulin. Moreover, bacteria exert an influence on TJ proteins, leading to an increase in the permeability of polarized epithelial cells. Noteworthy, it is evident that the activation of TLR2 by staphylococcal enterotoxin can potentially undermine the structural integrity of TJs and the epithelial barrier through the induction of pro-inflammatory cytokines. The purpose of this article is an attempt to investigate the possible role of the most important microorganisms associated with CRS and their pathogenic mechanisms against mucosal surfaces and epithelial barriers in the paranasal sinuses.

Video Abstract

**Supplementary Information:**

The online version contains supplementary material available at 10.1186/s12964-023-01347-2.

## Introduction

Chronic rhinosinusitis (CRS) is a disease that causes inflammation in the upper airways and sinuses and lasts for at least 12 weeks. Depending on whether nasal polyps (NPs) are present, CRS can be classified into two phenotypes: CRS without NPs (CRSsNP) and CRS with NPs (CRSwNP). Type 1 inflammation and type 2 inflammation characterize CRSsNP and CRSwNP, respectively [1, 2]. Due to CRS’s great degree of heterogeneity, its pathophysiology is still unclear. Different factors, such as racial and geographical factors, the dysfunction of mucociliary clearance (MCC), destruction of epithelial barriers, disrupted immune response, biofilm community of microorganisms, and dysbiosis of sinus microbiota, be related to the onset and development of CRS [3, 4].

The mentioned factors can stimulate nasal epithelial cells to produce epithelial-derived cytokines. Eosinophilic infiltration and a T-helper 2 (Th2)-based cytokine profile were found in nearly 80% of CRSwNP. However, the CRSsNP was more strongly connected with categories 1 and 3 of inflammation, which are characterized by elevated levels of TNF-α, IL-6, IL-8, and IL-17 [5]. The prevalence of microorganisms like fungi, bacteria, and viruses within the nose and paranasal sinuses in CRS patients has been widely established, although the precise cause of the inflammation associated with this chronic illness condition is still unknown [6, 7].

Recently published studies have reported that bacterial infections are linked to both acute and CRS. To this end, the most prevalent respiratory infections found in patients with acute sinusitis are *Staphylococcus aureus*, *Moraxella catarrhalis*, and *Streptococcus pneumoniae* [8]. With a higher prevalence of coagulase-negative staphylococci (CONS), *S. aureus*, Gram-negative bacteria, and anaerobic bacteria, CRS has a different microbiological pattern than acute sinusitis [6]. Patients who have had prior sinus surgery have also been demonstrated to have greater *Pseudomonas aeruginosa* prevalence rates [9, 10]. Collectively, the possibility exists that the colonization of these bacteria by the nose or paranasal sinuses could result in CRS, prolong the illness, or exacerbate a noninfectious inflammatory process [6].

So, it is thought that bacteria may contribute directly or indirectly to the development or persistence of CRS and clinical practice guidelines for its treatment show that most medical professionals agree that the presence of bacteria is a major factor in the pathogenesis and cause of many CRS cases. In actuality, doctors frequently recommend antimicrobial therapy for the treatment of CRS in a variety of ways [6, 11].

Therefore, as mentioned, CRS is a multifactorial disease, and microorganisms could play a critical role in the progression of this disease. In this regard, the possible role of the microorganism has been reported in previous research. For instance, *S. aureus* is a common human bacterium that is often found in the usual microbiota of the noses of healthy people. *S. aureus* enterotoxin B (SEB) was linked to CRS and nasal polyps as a risk factor. Chronic inflammation and damage to the inside of the nose caused by SEB are the main causes of CRS. In this way, an earlier study found that *S. aureus* was present in the noses of 67% of CRSwNP patients [12]. This bacterium can colonize the nasal mucosa under specific circumstances, which may facilitate its invasion into the subepithelial areas [13]. The functions of eosinophils and mast cells are improved by SEB, and the production of Th2 cytokines is increased. In addition, SEB can induce reactive oxygen species (ROS) and endoplasmic reticulum stress in the epithelial cells of patients with CRSwNP [14, 15].

*P. aeruginosa* is an additional prevalent bacterial species observed in non-cystic fibrosis CRS patients, and its existence is linked to the manifestation of severe and persistent CRS [16]. *P. aeruginosa* infections and the biofilm community of this bacterium have been implicated in recalcitrant CRS that contribute to inflammation [17]. Moreover, there exists a correlation between fungal allergens and dysregulation of the immune response, malfunction of the epithelial barrier, intensification of local inflammatory reactions, and CRS [18]. One of the most commonly found fungus species in nasal discharge is *Alternaria alternata*. This particular species has been observed to exhibit strong immunologic activity in nasal epithelial cells, suggesting its potential significance in the development of chronic CRS. Furthermore, the interplay between this fungal organism and the host’s innate and adaptive immune responses gives rise to the onset and progression of persistent inflammation. The resulting inflammation may subsequently initiate CRS and the development of nasal polyps [19]. The inhibition of surfactant protein synthesis or intracellular reserves and the excessive production of mucus could impede the elimination of *Alternaria* from the sinuses and perhaps contribute to the colonization and re-infection by airborne fungi [20].

Therefore, the interactions between microorganisms and the immune system could lead to CRS. Furthermore, the destruction of mucosal barriers, epithelial remodeling, and impaired mucociliary clearance are other possible pathogenic mechanisms of microorganisms in CRS. To this end, the present review paper is an attempt to investigate the possible role of the most important microorganisms associated with CRS and their pathogenic mechanisms against mucosal surfaces and epithelial barriers in the paranasal sinuses.

### Mucosal barriers and mucociliary clearance activities against microorganisms

The sinonasal tract serves as a location where the external world interacts with the body. This interaction involves the encounter and subsequent removal of foreign antigens through mechanisms such as MCC, as well as specific and generic immune responses [21]. The nasal mucosa is protected by the epithelium, which serves as the initial barrier against potential threats. The fundamental mechanical barrier is formed by cell-cell contact and the MCC system [3].

The MCC system consists of three fundamental functional components: mucin production, cilia activity, and the airway surface liquid layer. The primary role of ciliated epithelial cells is to facilitate the removal of diverse microorganisms and inhaled irritants that become entrapped within the airway surface fluids and mucus. Several previous investigations have documented a relationship between CRS and a decline in MCC, with the extent of this decline being positively associated with the severity of CRS [22].

The nasal mucosa is constantly exposed to a variety of microorganisms such as bacteria (in the form of biofilm and superantigens), viruses, fungi, and non-living foreign proteins [23]. It is worth mentioning that individuals diagnosed with CRS frequently have a localized infection within the nasal and paranasal cavities. Various microorganisms, including *S. aureus, H. influenzae*, *Aspergillus fumigatus, S. pneumoniae*, and *P. aeruginosa*, have been seen to secrete diverse toxins that possess the ability to efficiently eradicate ciliated epithelial cells [24]. Mucociliary dysfunction has been identified as a contributing element to the pathogenesis of CRS. This dysfunction compromises the protective capabilities of the nasal mucosa, making it more susceptible to bacterial attachment and biofilm formation. These findings highlight the significance of mucociliary dysfunction in the pathogenesis of CRS [3].

The physical-mechanical barrier is comprised of many cellular structures, including adherence junctions, gap junctions, tight junctions (TJs), desmosomes, and hemidesmosomes. The aforementioned components play a crucial role in promoting robust cell-cell adhesion, creating a highly selective barrier, establishing cellular polarity, and effectively modulating the movement of various molecules and ions [25]. TJs are composed of many proteins, including zonula occludens-1 (ZO-1), claudins, junctional adhesion molecule 1 (JAM-1), and occludin. Adherens junctions are comprised of transmembrane proteins, namely nectin and E-cadherin, as well as intracellular proteins α- and β-catenin. Desmosomal attachments are formed through the heterotypic binding of desmoglein molecules [26, 27].

When the integrity of the epithelial barrier in the sinuses is compromised, there is a significant transformation in its structure and function. It transitions from being a passive physical barrier to becoming an active organ with the ability to secrete various bioactive substances, including cytokines and complement proteins. This transformation enables the recruitment and regulation of diverse immune cells [28, 29]. In this regard, the sinonasal epithelium is known to have a significant impact on both innate and adaptive immunity. Hence, the atypical functionality of the epithelium can exert a substantial influence on the initiation and progression of CRS [3].

In the normal individual, the mucosal immune system exhibits a response to stimulation by serving as an initial barrier against invading pathogens, thereby mitigating the occurrence of excessive tissue damage and inflammation as observed in CRS [30]. The destruction of the epithelium results in an increase in epithelial permeability. The introduction of external irritants can consequently stimulate the activation of immune cells, thereby initiating an immunological response [31]. There have been two separate but interconnected responses identified in the context of microbial pathogens and foreign proteins, namely innate and acquired immunity. The respiratory epithelium, which constitutes a physical barrier with TJs, serves as the initial line of defense and is a significant constituent of the innate immune system within the nasal cavity. Additionally, enzymes and peptide antibiotics are excreted inside mucus, exerting direct antibacterial action [32].

The subsequent line of defense is comprised of neutrophils and macrophages, which engage in the process of phagocytosis against microbial invaders. The presence of barrier abnormalities and diminished local innate immunity can potentially trigger the immunological and inflammatory reactions observed in CRS [23, 33]. Recent investigations have delineated three distinct endotypes of CRS based on the increased levels of classical T-cell cytokines in this pathological state. Type 1 is primarily linked to an elevation in Th1 cytokines, specifically TNF-α and IFN-γ. Type 2 is characterized by an upregulation of Th2 cytokines, namely IL-4, IL-5, and IL-13. Type 3 is associated with the Th17 cytokines. Upon exposure to exogenous irritants, nasal epithelial cells have been seen to release a diverse array of inflammatory cytokines, including but not limited to TNF-α, IL-25, thymic stromal lymphopoietin (TSLP), IL-33, and IL-6. There is a prevailing consensus that CRSsNP mostly encompasses Th1-type inflammation and has elevated levels of IFN-γ expression [34].

Epithelial-derived cytokines, namely IL-25, IL-33, and TSLP, play a crucial role in promoting the Th2 response during the formation of innate lymphoid cells (ILCs). These cytokines also stimulate the production of Th2 cytokines, including IL-4, IL-5, and IL-13, which in turn contribute to the amplification of type 2 inflammation [35]. IL-6 is responsible for promoting the differentiation of naive T-cells into Th17 cells and facilitating the production of IL-17, a type 3 cytokine that plays a crucial role in type 3 inflammation. [3]. Epithelial barrier impairments can be caused by the release of pro-inflammatory cytokines such as IFN-γ and IL-4. These cytokines can break TJs between epithelial cells and thus decrease the resistance of the epithelial barrier to the movement of substances across it [36].

There is evidence suggesting that neutrophils may have a detrimental effect on epithelial barrier function. This effect is thought to be mediated by the release of oncostatin M, a cytokine known to alter the integrity of the epithelial barrier [37]. The excessive production of mucus is a characteristic feature of CRS. The upregulation of Th2-mediated pendrin expression could potentially play a role in the augmentation of mucus production and the reduction of mucociliary clearance in patients with CRSwNP [38]. Noteworthy, transepithelial electrical resistance (TER) serves as an indication that reflects the functionality of the epithelial barrier [39]. Patients diagnosed with CRSwNP display a decrease in TER, which may potentially be linked to impairment of the epithelial barrier [36].

Finally, in the airway, the presence of impaired epithelial tissue is a significant factor in initiating the process of tissue remodeling [40, 41]. Tissue remodeling refers to the anomalous repair or restitution of injured tissue in response to inflammation or mechanical injury, which can manifest in any organ. Remodeling, a prominent characteristic of CRS, encompasses many layers of sinonasal tissues, namely the epithelium, sub-epithelium, and underlying bone [41]. Within the context of CRS, studies have documented many forms of tissue remodeling, such as basement membrane thickening, mucosal hypertrophy, angiogenesis, fibrosis, collagen deposition, and osteitis [42]. Mucosal remodeling has been observed in both CRSwNP and CRSsNP. Additionally, it has been noted that eosinophilic CRS is correlated with significant eosinophilic infiltration in the nasal mucosa [43, 44]. The available evidence indicates a potential correlation between eosinophil activation and eosinophilia and CRS remodeling and mucosal damage [45].

Furthermore, an in vitro study has discovered that the release of eosinophil-derived neurotoxin from eosinophils, which are stimulated by IL5, can significantly enhance the production of matrix metalloproteinase 9 by the nasal epithelium. This process has the potential to impact the regeneration of the epithelial tissue and the degradation of the extracellular matrix (ECM), ultimately resulting in nasal remodeling [46]. ECM proteins have a crucial role in the preservation of structural support, maintenance of physiological balance, and modulation of inflammatory processes inside the airway mucosa. The expression of periostin, an ECM protein, is upregulated by IL-4 and IL-13, leading to its secretion by airway epithelial cells [42, 47].

It is noteworthy that research investigations focusing on nasal mucosal biopsies of CRS have revealed that periosteal proteins can induce eosinophilic infiltration and facilitate fibrosis, thereby playing a role in the process of mucosal remodeling [48]. Transforming growth factor-beta (TGF-β) is a growth factor with pleiotropic and multifunctional properties, capable of stimulating fibroblast proliferation, differentiation, and the development of fibrotic traits. Furthermore, it can induce the synthesis of tissue inhibitors of metalloproteinase 1 (TIMP-1), thereby impeding the enzymatic degradation of ECM [49]. According to the available reports, a notable disparity has been seen between CRSsNP and CRSwNP regarding the levels of TGF-β. It has been found that CRSsNP exhibits elevated TGF-β levels, accompanied by the presence of denser collagen fibers in the ECM. Consequently, this molecular milieu contributes to an exaggerated tissue healing response and the development of fibrotic tissue. In contrast, the presence of TGF-β is lacking in CRSwNP, resulting in poor tissue repair. This is evidenced by the occurrence of loose connective tissue and the production of edema in the significantly inflamed tissues [50].

Nevertheless, the precise pathomechanism responsible for tissue remodeling remains unclear and is currently the subject of ongoing research efforts. Collectively, as mentioned, mucosal barrier disruption, inhibition of mucociliary function, and mucosal remodeling are important factors in CRS. To this end, in the next sections, we will discuss the possible role of microorganisms in the production of the mentioned factors.

### *P. aeruginosa*

Infections caused by *P. aeruginosa* have a significant death rate due to their high antibiotic resistance. This bacterium induces significant tissue damage through a range of virulence factors, and its ability to produce biofilms leads to the development of persistent infections that are resistant to antibiotics [51]. The findings of recent research have demonstrated that individuals diagnosed with CRS exhibit a greater abundance of this bacterium as compared to people without CRS. The observed variation in bacterial characteristics could potentially be linked to the progression of diseases or the ability to resist routinely employed antibiotics through mechanisms of resistance and the production of biofilms [52]. To this end, finding the exact role of *P. aeruginosa* in the initiation or progression of the CRS has been considered by researchers in recent years.

As mentioned in the previous parts, CRS is a complex condition characterized by a multifactorial etiology, which leads to a pronounced inflammatory reaction in the affected area and compromised functionality of the MCC mechanism. Noteworthy, the functioning of cilia plays a crucial role in the defense mechanism of the upper airways, where dysfunction is observed in chronic conditions [53]. In this concept, *Shen* et al., for the assessment of sinonasal ciliary function, administered bacteria supernatant, including *S. aureus*, *H. influenza*, *P. aeruginosa*, and *S. pneumoniae*, to the murine primary sinonasal cultures that were established in an air-liquid interface (ALI). All of the bacteria supernatants decreased basal ciliary beat frequency (CBF); however, *P. aeruginosa* and *S. pneumoniae* caused a remarkable decrease in CBF. Furthermore, *P. aeruginosa* led to a reduction in the ciliary stimulation capacity [9]. Therefore, *P. aeruginosa* could enhance the chance of CRS by reducing basal CBF; however, the exact role of this bacterium in this process is unknown, and further studies are required in the field.

In addition to CBF inhibition, one potential mechanism by which *P. aeruginosa* may contribute to CRS is the disruption of the mucosal barrier via the release of secreted compounds that contribute to the inflammatory response. Disruption of TJs has been observed in various chronic illnesses, such as CRS. In nasal polyp epithelial cell cultures, a diminished expression of TJ and adhesion junction proteins, including occludin, ZO-1, claudin-1, and E-cadherin, has been seen in comparison to healthy mucosa [17, 54, 55]. The failure of the nasal barrier has the potential to cause an elevation in permeability to allergens such as house dust mites. This, in turn, can contribute to the development of allergic sensitization and subsequent mast cell degranulation. The release of cytokines and pro-inflammatory factors, including histamine, INF-γ, and IL-4, in response to infection or inflammation can lead to barrier dysfunction. This dysfunction can potentially exacerbate the involvement of *P. aeruginosa* in asthma or CRS through indirect mechanisms [56, 57].

In this regard, in a recently published study, the authors applied *P. aeruginosa* exoprotein to the ALI cultures of primary human nasal epithelial cells (HNECs-ALI). Different methods, such as immunofluorescence of TJ proteins, the passage of FITC-dextrans, and transepithelial electrical resistance (TEER), were used for the evaluation of mucosal barrier integrity. The P. *aeruginosa* exoprotein remarkably increased the production of IL-6 and led to the disruption and relocalization of the TJ proteins. Moreover, the enhanced permeability of FITC-dextrans and the decreased TEER provided further evidence of barrier rupture. Interestingly, the detrimental impact exhibited a reversible nature and was effectively counteracted with the application of proteinase K [17].

In line with these results, *Kao* et al. also reported that serine proteases originating from *P. aeruginosa* and neutrophils have adverse impacts on the integrity of the mucosal barrier. This leads to heightened permeability, which in turn facilitates the possibility of bacterial invasion [53]. In addition to serine proteases, the findings of another investigation indicated a significant correlation between *P. aeruginosa* elastase activity and mucosal barrier disruption, as evidenced by increased permeability of FITC-dextrans and decreased TEER [58].

This supports the finding by *Nomura* et al., who reported that the elastase of *P. aeruginosa* leads to the transient disruption of TJ and downregulation of protease-activated receptor-2 (PAR-2) in human nasal epithelial cells. It’s noteworthy to mention that PARs have extensive cellular expression inside several bodily structures, including connective tissue, blood vessels, leukocytes, epithelium, and numerous airway cells. Furthermore, the activation of PAR-2 has been observed to have an impact on the integrity of the airway epithelial barrier. The results of this study showed that the elastase destroyed the epithelial barrier and reduced the transmembrane proteins tricellulin, occludin, and claudin-1 and − 4. On the other hand, this bacterial enzyme did not show a significant effect on the scaffold PDZ-expression proteins ZO-1 and − 2 and adherence junction proteins β-catenin and E-cadherin. Moreover, *P. aeruginosa* elastase reduced the expression of PAR-2, which also regulated the expression of the TJ proteins [59].

The disruptive effect of elastase on the mucosal barrier has been evidenced by its ability to cause the reorganization of TJ proteins. Moreover, elastase possesses the capacity to impede pro-inflammatory reactions by cleaving thrombin and liberating C-terminal-derived peptides. The release from this peptide binds to pathogen-associated lipopolysaccharide, hence inhibiting cellular activation and subsequent proinflammatory reactions [59, 60].

**Fig. 1:**
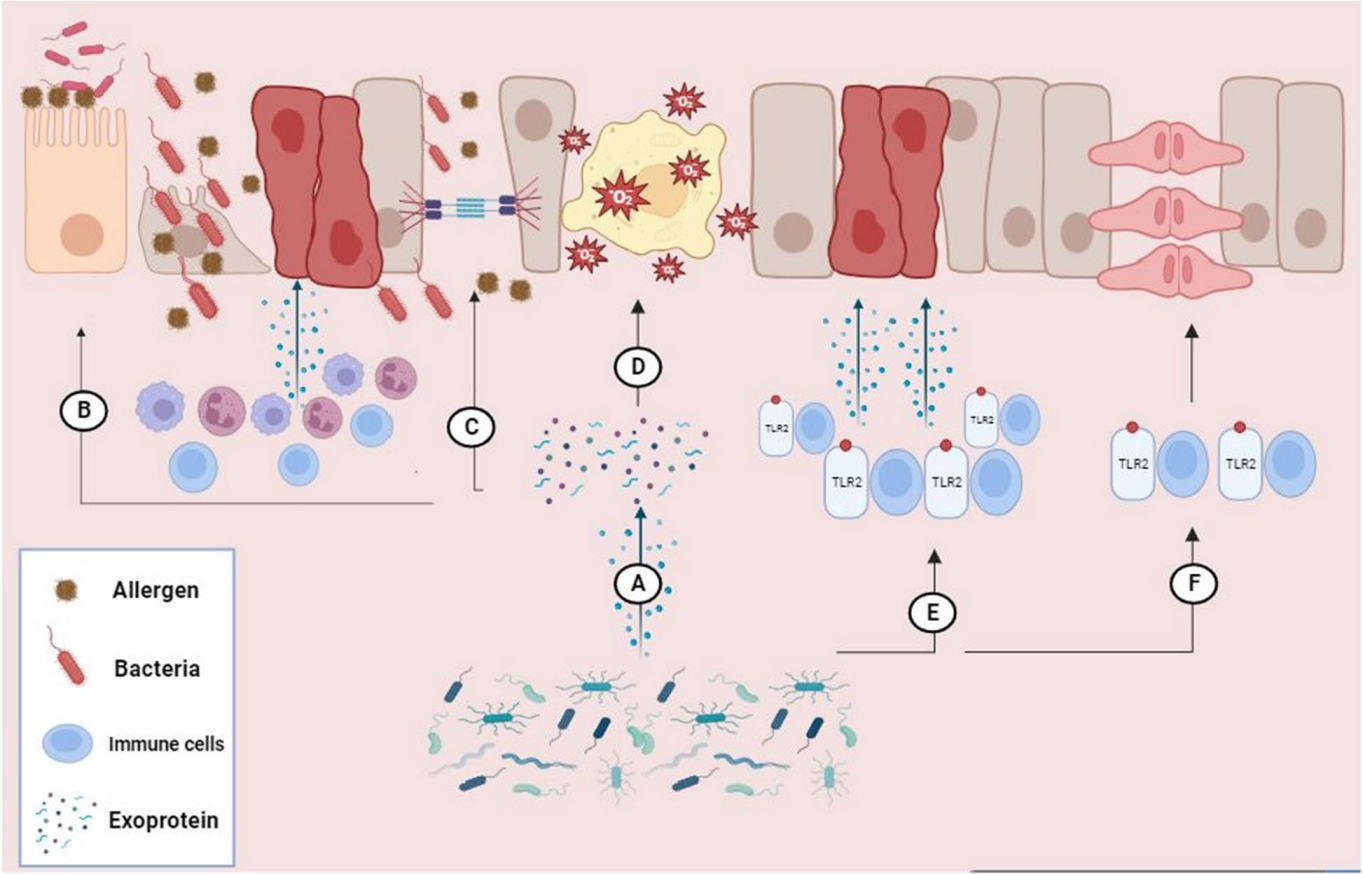
Possible pathogenic mechanisms of bacteria in CRS. **A** bacteria produce exoproteins and play a critical role in the pathophysiology of CRS through **B** severe disruption of ciliary functions and **C** both the cytoskeleton and apical junctional complex proteins. **D** Reactive oxygen species generated by bacterial toxins have the potential to disrupt the normal functioning of epithelial cells and lead to alterations in their morphology. Moreover, **E** the bacterial toxin could activate TLR2 and lead to the production of inflammatory cytokines, thereby decreasing epithelial integrity. **F** Finally, colonization or exposure by bacteria in the airway could increase tissue remodeling

The aforementioned virulence pathways potentially play a role in the persistent and recalcitrant nature of *P. aeruginosa* infections in CRS. Hence, as previously said, the exoproteins of this bacterium, particularly elastase and serine protease, have been demonstrated to disrupt the epithelial barrier and induce downregulation of transmembrane proteins such as occludin, claudin-1, claudin-4, and tricellulin. Moreover, *P. aeruginosa* exerts an influence on TJ proteins, leading to an increase in the permeability of polarized epithelial cells. Therefore, although further investigation is required to elucidate the precise mechanistic impacts of *P. aeruginosa* exoproteins on the integrity of the epithelial barrier, the exoproteins of this bacterium could play a critical role in the pathophysiology of CRS by significantly disrupting ciliary functions and both the cytoskeleton and apical junctional complex proteins. Additionally, these exoproteins should be considered as potentially important therapeutic targets for inhibition and management of CRS (Fig. 1).

### *S*. *aureus*

According to recent investigations, it has been observed that nasal colonization of *S. aureus* was identified in 67% of patients diagnosed with CRSwNP. Additionally, it has been shown that nearly 50% of nasal tissue homogenates obtained from nasal polyps contain specific IgE antibodies against *S. aureus* enterotoxins [5]. Therefore, previous studies have brought attention to the significance of staphylococcal virulence factors in the development of CRSwNP. One of the main pathogenic mechanisms associated with *S. aureus* in CRSwNP is the disruption of mucosal integrity. In this regard, in this section, we will discuss the possible role of S. *aureus* in CRS via epithelial cell integrity disruption and enhancing their permeability.

*Panchatcharam* et al. reported that *S. aureus* biofilm exoproteins are toxic and disrupt the mucosal barrier structure in a time- and dose-dependent manner [61]. Another study also reported that *S. aureus* strain 13,565-secreted products could damage the airway epithelium by disrupting the TJs between primary HNECs-ALI [62]. In line with these results, *S. aureus* strain 13,565 was also used in another investigation, and the authors suppose that *S. aureus* is responsible for TJ disruption in HNEC-ALI cultures as either a protein-macromolecule or a combination of secreted factors [63, 64]. Noteworthy, *S. aureus* strain 13,565 which severely compromised the TJ structure is known to produce enterotoxin A24 and hemolysin B [63]. Finally, *Murphy* et al. investigated the impact of the purified *S. aureus* V8 protease, a 29 kDa serine protease, on airway epithelial integrity. The results showed that the application of V8 protease to the sinonasal cell layer results in a notable impact on the TJ barrier. This is characterized by a decrease in the protein ZO-1, while the protein claudin-1 experiences relatively minor changes. The paracellular permeability is significantly altered, and there is a fragmentation of protein localization. Noteworthy, the alterations seen in ZO-1 may be attributed to either protein redistribution or degradation. This phenomenon could be a result of proteolytic degradation or an indirect mechanism [55]. Hence, the mentioned virulence factors may be associated with mucosal barrier dysfunction; however, more confirmatory research should be conducted.

Noteworthy, the recently published study investigated the role of staphylococcal enterotoxin B (SEB) in the pathogenesis of CRS. To this end, in their study, *Martens* et al. utilized SEB to treat the nasal polyp epithelial cells of patients with CRSwNP. The findings of their investigation revealed that SEB administration led to an increase in FD4 permeability and a decrease in TEER, with the effects being dependent on the dosage of SEB. In general, the presence of SEB leads to the disruption of the integrity of epithelial cells, as well as the inhibition of the expression of occludin and ZO-1 proteins. Additionally, the activation of TLR2 by SEB resulted in the synthesis of IL-6 and IL-8. The application of these cytokines to an air-liquid interface culture resulted in a reduction in epithelial integrity. Significantly, the prevention of SEB-induced barrier disruption was shown through the reduction of TLR2 signaling. The outcomes of the animal model study further validate these results. To this end, the researchers administered 50 µl of SEB (Endonasal) to the nasal cavity of the control mice and afterward noticed that SEB had a detrimental effect on the integrity of the barrier function. Additionally, the expression of occludin and ZO-1 mRNA was shown to be reduced. However, the aforementioned parameters exhibited no changes in mice lacking the TLR2 gene [62].

Additionally, the interaction of SEB and the endoplasmic reticulum (ER) stress response was also considered a possible mechanism of CRS. Disturbances in the maintenance of ER homeostasis, such as increased protein synthesis, impaired ER redox balance, and the accumulation of misfolded proteins, have the potential to induce the ER stress response. In addition, it should be noted that inflammation plays a significant role in the generation of ROS, the induction of ER stress, and the progression of nasal polyposis [65]. In this concept, the findings of a study indicated that membrane-derived vesicles have the potential to transport SEB into the cytoplasm of epithelial cells, potentially leading to the activation of the ER stress response mediated by SEB [66]. SEB is recognized as a crucial mediator of inflammation in human nasal epithelial cells. As previously stated, a correlation exists between inflammation and the ER stress response, which leads to either tissue healing or the regulation of tissue damage. Nevertheless, the ultimate effect of inflammation generated by ER stress can be either deleterious or protective [67, 68].

Hence, the authors postulated that the induction of ER stress by SEB could potentially be associated with the advancement of nasal polyposis. Furthermore, it has been observed that SEB stimulates the generation of ROS in both eosinophilic and non-eosinophilic polyps, in contrast to the unaffected healthy mucosa. The present discovery indicates that ROS generated by SEB could potentially play a role in ER stress responses [66]. Notably, certain allergens and environmental pollutants can induce the ER stress response and the generation of ROS, which can ultimately lead to mitochondrial dysfunction in airway epithelial cells [69].

Noteworthy, ROS are molecular entities that possess the ability to exist autonomously, characterized by the presence of at least one oxygen atom and one or more unpaired electrons. ROS were initially identified as deleterious byproducts generated during the process of aerobic metabolism. Under normal physiological circumstances, minimal amounts of ROS are generated during cellular activities, such as aerobic respiration or inflammatory responses, primarily within hepatocytes and macrophages. ROS serve as key signaling molecules and are also involved in muscular contractions, the regulation of vascular tone, as well as the determination of bactericidal and bacteriostatic activity. The elevation in the generation of free radicals can be attributed to excessive exposure to ultraviolet (UV) radiation, prolonged periods of stress, rigorous physical activity, inadequate dietary habits, and the consumption of stimulant substances. Under normal physiological circumstances, there exists a state of equilibrium wherein the production and elimination of free radicals within the human body are balanced [70, 71].

Mitochondrial failure and increased levels of ROS have been documented in a diverse range of allergy conditions, including atopy, asthma, allergic rhinitis, and tissue damage observed in asthma. This includes detrimental effects such as epithelial cell impairment, shedding of cells, and heightened airway hyperresponsiveness [72]. The presence of environmental contaminants and allergens has been observed to result in mitochondrial dysfunction in airway epithelial cells through the stimulation of ROS generation and the ER stress response. Mitochondrial reactive oxygen species (mtROS) are generated as a consequence of oxidative phosphorylation occurring inside the mitochondrial electron transport chain. Mitochondria has the capacity to regulate ROS through the action of manganese-dependent superoxide dismutase (Mn-SOD), an enzyme that acts as a scavenger of mitochondrial ROS (mtROS). Nevertheless, the functionality of Mn-SOD can be impeded by a range of conditions, such as diverse infections and the inhalation of cigarette smoke, resulting in the generation of ROS [15, 72, 73]. Collectively, the process of oxidative phosphorylation within the electron transport chain of mitochondria leads to the generation of mtROS. The regulation of mtROS is carried out by mitochondria through the utilization of Mn-SOD. However, the activity of Mn-SOD is reduced and the formation of ROS is increased by cigarette smoke and exogenous infections [74, 75].

In this respect, *Yoon* et al. observed an elevation in mtROS levels in a human nasal epithelial cell line following exposure to SEB, which was found to be associated with the development of nasal polyps [76]. Actually, mtROS have the potential to modulate both the structure and function of mitochondria, leading to the disruption of normal cellular processes and the development of pathological conditions [77]. Collectively, it is evident that the activation of TLR2 by SEB can potentially undermine the structural integrity of TJs and the epithelial barrier through the induction of pro-inflammatory cytokines. This event leads to the loss of epithelial cell integrity and increases their permeability. In this context, the potential therapeutic strategy of targeting the TLR2 signaling pathway presents itself as a promising avenue for mitigating the pathophysiological effects of SEB on inflammation in CRSwNP.

Additionally, the administration of SEB has been seen to stimulate the production of ROS and trigger ER stress-induced inflammation. These processes have the potential to disrupt the normal functioning of epithelial cells and lead to alterations in their morphology. However, additional animal and clinical investigations are required to precisely ascertain these mechanisms. Hence, the exoproteins produced by *S. aureus* have the potential to induce dysfunction in the sinonasal epithelium’s barrier, disrupt mucus secretion thereby compromising the innate barrier, enhance exposure to antigens, and commence infection in the subepithelial region. Exoproteins are responsible for the manifestation of staphylococcal virulence and the initiation of inflammation. It should be noted that this phenomenon is not limited to the impact of a solitary toxin. In this context, it is postulated that a collection of proteins, including enterotoxins, hemolysins, and proteases, may be involved in the observed inflammation. However, it is important to note that only a subset of these proteins is likely to be responsible for the actual breakdown of the barrier [61].

*S. aureus* superantigens, specifically SEA and SEB, can enhance chronic inflammation in CRSwNP by stimulating a significant population of T cells. The activation of TLR2 by SEB leads to the production of pro-inflammatory cytokines. Additionally, SEB stimulates the generation of ROS and inflammation caused by ER stress. These processes have the potential to disturb the integrity of epithelial cells and increase their permeability [5].

Additionally, lipoteichoic acid (LTA) is a major component of the Gram-positive cell wall, which is composed of polymers consisting of repeated phosphodiester-linked polyols located in the outer plasma membrane [78]. The adhesion molecule LTA functions by binding to both TLR2 and the CD14 receptor, leading to the subsequent release of proinflammatory cytokines, reactive nitrogen, and oxygen species, as well as antimicrobial peptides [79]. To this end, the activation of TLR2 by LTA in lung endothelial cells is responsible for the induction of an elevated permeability of the cellular barrier. This effect is achieved by the production of ROS and reactive nitrogen species [64].

Finally, the soluble secreted protein known as alpha-hemolysin (Hla) engages in interactions with eukaryotic cell membranes. The monomeric form of Hla exhibits binding affinity towards the plasma membrane, leading to the formation of a heptameric transmembrane pore. There have been reports indicating a decrease in ciliary activity and the presence of ultrastructural alterations in nasal airway cells in vitro [64, 80]. Collectively, different exoproteins of *S. aureus* could lead to disruption of the mucosal barriers and increase the chance of CRS. Noteworthy, the interactions of *S. aureus* with mucosal barriers were also shown in an animal model [81]; however, data in this field is not complete and additional studies are required to definitively exclude any contribution to barrier dysfunction.

In the end, it is noteworthy to mention that *S. aureus*, in addition to disrupting barrier integrity, could lead to epithelial-mediated remodeling of allergic airways. Tissue remodeling occurs in the airway tissue of individuals with respiratory diseases and is characterized by augmented collagen deposition, hyperplasia of smooth muscle and submucosal glands, as well as fibrosis [82]. One of the significant discoveries in the advanced remodeling tissue of asthma patients is the presence of an imbalance between matrix metalloproteinases (MMPs) and tissue inhibitors of metalloproteinases (TIMPs) at the molecular level.

To this end, *Homma* et al. conducted an experiment including the stimulation of normal human bronchial epithelial (NHBE) cells with transforming growth factor (TGF)-α and *S. aureus*. The bacteria elicited the production of both mRNA and protein for TGF-α and MMP 1 from NHBE cells through a TLR2-dependent pathway [83]. In summary, *S. aureus* can activate TLR2, resulting in the significant induction of MMP-1 expression in primary airway epithelial cells. Therefore, the presence or colonization of this bacterium in the respiratory tract may contribute to tissue remodeling by inducing the production of MMP-1 through a TGF-α-dependent mechanism. This process could potentially contribute to the development of airway illnesses, such as asthma and CRS. Nonetheless, we could not find confirmatory data on this subject; therefore, more confirmatory studies are needed.

It should be noted that, in addition to *P. aeruginosa* and *S. aureus*, other microorganisms such as Streptococcus pneumoniae, *Alternaria alternata*, *rhinoviruses*, *Aspergillus*, and *Haemophilus influenza could* also disrupt the mucosal barrier and increase the chance of CRS. The data about the mentioned microorganism is limited; however, the funded article is presented in Table 1.

**Table 1:** The role of other microorganisms in chronic rhinosinusitis and destruction of epithelial surfaces and mucosal barriers

Year of publication	Microorganisms	In vitro/In vivo(methods)	Result	References
2019	*Aspergillus fumigatus*, *Alternaria alternata*, *Staphylococcus aureus*	Animal model	The induction of eosinophil infiltration and epithelial remodeling was observed as a result of prolonged exposure to airborne allergens.	[84]
2018	*Alternaria*	Western blot analysis, Real-time reverse transcriptase-PCR, and confocal microscopy.	The nasal epithelial barrier function was modified by the presence of serine protease in *Alternaria*. The presence of intracellular ROS triggered by *Alternaria* has the potential to impact the function of the barrier of nasal epithelial cells and intensify the inflammatory processes occurring in the nasal mucosa.	[85]
2008	*Aspergillus versicolor* *Alternaria alternata*	Animal model	After exposure to microorganisms, non-invasive fungal sinusitis had been induced with a rise in neutrophil cluster counts. The hyperplasia of goblet cells, increased mucus production in the sinonasal cavity, inflammatory cell infiltration, and epithelial thickening were all seen in animals with fungal sinusitis.	[86]
2016	*Aspergillus flavus*	ALI cultures of bronchial cells and primary human sinonasal	Aflatoxins decreased baseline and agonist-stimulated CBF. Therefore, *A. flavus* aflatoxin has the potential to hinder the motile and chemosensory capabilities of airway cilia. This impairment has an important function in the progression and development of fungal airway illnesses.	[87]
2013	*Aspergillus fumigatus* *Staphylococcus aureus, Pseudomonas aeruginosa*, *Haemophilus influenzae, Staphylococcus epidermidis*	Sheep sinusitis model, CLSM. Transmission electron microscopy and light microscopy were each used to evaluate the integrity of the cilia and the degree of inflammation.	Bacterial biofilms have been identified as a contributing factor to the development of sinonasal mucosal inflammation and epithelial damage, creating an environment conducive to the expansion of fungal biofilms. Enhancing postoperative cilial recovery and managing bacterial biofilms are crucial factors in mitigating the persistent nature of allergic fungal rhinosinusitis in patients.	[88]
2018	*Influenza A virus*	In vitro hNEC model	Viral infection remarkably enhanced OSM expression. Noteworthy, the disruption of TJs may be caused by increased OSM expression, which is implicated in CRSwNP and may be amplified by influenza infection.	[89]
2020	*Rhinovirus*	Epithelial markers and TJ proteins were examined using in vitro IL-13-matured hNECs and nasal samples from patients with CRSwNP.	Following IL-13 therapy, CRSwNP biopsies and hNECs showed considerably lower levels of occludin, ZO-1, claudin-3, and expression. The administration of IL-13 resulted in an elevation in the permeability of the epithelium, a reduction in TEER, and a modification in the composition of hNECs. These changes were associated with a decrease in the number of ciliated cells and excessive production of mucus. Although RV infection had limited effects on TJs, it was observed that human nasal epithelial cells (hNECs) matured with IL-13 had a decreased ability to upregulate IFN-λ1 and CXCL10 in response to RV infection. However, the expression of(TSLP was further elevated following RV infection in these IL-13-matured hNECs. The results of this study indicate that IL-13 is associated with impaired tight junctions and decreased integrity of the epithelial barrier. The loss of cilia produced by IL-13 resulted in reduced viral replication and compromised antiviral responses of the nasal epithelium to RV infection.	[90]
2015	*Rhinovirus*	Real-time PCR, immunoblot analysis, immunohistochemistry	The expression of Th2-mediated pendrin is elevated in nasal polyps seen in patients diagnosed with CRS. This heightened expression has the potential to induce inflammation, increased production of mucus, and a reduction in mucociliary clearance. The presence of human rhinovirus was found to enhance the expression of pendrin triggered by IL-13.	[38]
2014	*Alternaria* *Aspergillus* *Rhinoviruses*	Real-time RT-PCR	The study examined the effects of fungi and RV-16 on the expression of mucin genes in nasal polyp epithelial cells. The presence of RV-16, airborne fungus, and eosinophils have the potential to intensify the inflammatory process in nasal mucosal disorders through the augmentation of mucin gene expression.	[91]

*hNECs *human nasal epithelial cells, *CRSwNP *Chronic rhinosinusitis with nasal polyps, *OSM *Oncostatin M, *E-CRS *Eosinophilic chronic rhinosinusitis, *PCR *Polymerase chain reaction, *CBF *Ciliary beat frequency, *TEER *Transepithelial electrical resistance, *RV* *Rhinovirus,* *TJs *Tight junctions, *ROS *Reactive oxygen species, *TSLP *Thymic stromal lymphopoietin, *ALI *Air-liquid interface, *CLSM *Confocal scanning laser microscopy, *ZO-1 *Zonula occludens-1

### Therapeutic approaches

As mentioned, the combined effect of *P. aeruginosa* exoprotein on enhancing mucosal permeability and degrading innate immune defense mechanisms may result in a persistent, yet inadequate, immune response. This inadequate response is a contributing factor to the development of severe inflammation and the production of polyps in individuals with *P. aeruginosa*-associated CRS. In this concept, understanding the interactions of microorganisms with mucosal surfaces and epithelial barriers in the paranasal sinuses can facilitate developing preventive and treatment approaches against CRSwNP. To this end, recently published studies used different approaches for inhibiting the destructive effects of microorganisms on mucosal surfaces and epithelial barriers in the paranasal sinuses.

*Lim* et al. used ciprofloxacin and azithromycin sinus stent (CASS) on human sinonasal epithelial cells (HSNECs) that had been stimulated by LPS from *P. aeruginosa.* Noteworthy, the CASS was specifically engineered to facilitate the controlled release of azithromycin and ciprofloxacin. This release is achieved through the utilization of two separate layers inside the system: an inner layer composed of hydrophilic ciprofloxacin and an outer layer composed of hydrophobic azithromycin. The administration of CASS therapy resulted in a notable reduction in the inflammatory response (IL-8) caused by LPS in HSNECs. This reduction was observed without any harmful effects on the cells and maintained the integrity and functionality of the epithelial layer. Actually, the administration of CASS did not result in any significant alterations in TEER, CBF, or paracellular permeability. These findings suggest that cellular integrity and functionality were preserved following treatment with CASS. Hence, the authors propose that the utilization of an antibiotic-eluting sinus stent for the continuous release of ciprofloxacin and azithromycin is a promising therapy approach with significant potential for enhancing clinical outcomes in CRS [92].

Additionally, the results of another study also indicated that Rhinosectan®, a medical device containing xyloglucan, contributed to the preservation of TJ, as demonstrated by an increase in TEER values across time. Noteworthy, Rhinosectan® did not alter the paracellular flux, even after treatment with LPS from *P. aeruginosa*. The findings of this study suggest that xyloglucan has the ability to inhibit P. *aeruginosa*-induced changes in TJ permeability [93]. To this end, the authors postulated that the existence of xyloglucan is believed to prevent the interaction between epithelial cells and aeroallergens, such as pollen, as well as the released cytokines, hence mitigating the allergic reaction. Furthermore, it is believed that preventing the interaction between bacterial LPS and the monolayers can reduce the inflammation of nasal epithelial cells caused by LPS. Consequently, this can mitigate the inflammatory response triggered by LPS derived from Gram-negative bacteria [93, 94].

Therefore, as mentioned, after recognizing bacterial interactions with mucosal surfaces and epithelial barriers in the paranasal sinuses, we can use a protective physical barrier on nasal epithelial cells and different agents to interfere with microbial invasion of the mucosal barriers. However, data in this field is very limited, and further studies are needed. In the end, it is noteworthy to mention that the mitigation of chronic inflammation in the sinonasal mucosa is a significant obstacle in the management of individuals diagnosed with CRS. The control of inflammation presents a potential avenue for the re-epithelialization of the lining of the sinonasal cavity as well as the restoration of functional cilia activity. In this concept, the presence of microbial-induced pro-inflammatory cytokines in the sinonasal mucosa is a significant contributing element to the degradation of mucosal barriers, hence increasing the susceptibility of individuals to CRSwNP. Anti-inflammatory agents such as bee venom, chitosan-dextran gel, prostaglandin E2, dexamethasone, and calcium channel blockers have been shown to inhibit microbial-induced pro-inflammatory cytokines. Therefore, future studies can consider these agents for inhibition of microbial destructive effects on mucosal surfaces and epithelial barriers in the paranasal sinuses [5, 95].

## Conclusion

The objective of this study was to examine the pathogenic mechanisms of common microorganisms in CRS and their involvement in the degradation of mucosal barriers, remodeling of epithelial tissue, and impairment of MCC. Bacteria, fungi, and viruses can exert a significant influence on the pathogenesis and advancement of CRS through the modulation of both innate and adaptive immune reactions. The role of epithelial disorder is crucial in the development or cause of CRS. The disruption of the epithelium barrier and failure of MCC are the primary mechanisms underlying the pathogenesis of CRS. The analysis of research findings has revealed that microbial-produced substances disrupt an essential element of mucociliary clearance. However, given the significance of various microorganisms in the etiology and exacerbation of this disease, as well as its profound implications for individuals’ well-being, further extensive investigation is imperative to substantiate the involvement of microorganisms in the destruction and disruption of epithelial barriers within the paranasal sinuses.

## Data Availability

The authors confirm that the data supporting the findings of this study is available within the article.
